# Discordance in Biomarker Expression in Breast Cancer After Metastasis: Single Center Experience in India

**DOI:** 10.1200/JGO.18.00184

**Published:** 2019-04-05

**Authors:** Ajay Gogia, S. V. Suryanarayana Deo, Dayanand Sharma, Rakesh K. Phulia, Sanjay Thulkar, Prabhat S. Malik, Sandeep Mathur

**Affiliations:** ^1^ All India Institute of Medical Science, New Delhi, India

## Abstract

**PURPOSE:**

Biomarker—estrogen receptor (ER), progesterone receptor (PR), and human epidermal growth factor receptor 2 (HER2/*neu*) —discordance plays an essential role in the management and prognosis of patients with metastatic breast cancer. Rates of discordance have been previously reported around 12% to 35%, 30% to 50%, and 5% to 15%, respectively, in Western literature. Data are sparse regarding the same from developing countries, such as India.

**METHODS:**

We performed an ambispective review of paired biomarker status in patients with breast cancer—stage I, II, and III as per American Joint Committee on Cancer, 7th edition—who developed metastasis at recurrence (N = 103 patients). Biomarker status and clinical and radiologic parameters were documented at baseline and subsequent follow-up.

**RESULTS:**

Discordance was present in 21.3% for ER, 29.1% for PR, and 15.5% for HER2/*neu* receptor. In our cohort, 7.8% had positive to negative ER and 13.6% negative to positive. Whereas 21.4% had positive to negative PR, 7.8% had negative to positive PR. Approximately 6.8% had positive to negative HER2/*neu* receptor and 8.7% negative to positive. In our cohort, 41 patients (40%) had single-site metastasis—bone, 15.5%; lung, 11.7%; nonregional lymph node, 7.8%; liver, 3.9%; and brain, 0.97%. More than one site of metastasis was present in 62 patients (60%). The most common sites of metastasis were visceral—lung and liver—followed by bone, nonregional lymph node, skin, and brain.

**CONCLUSION:**

The current study demonstrated that metastatic disease evolution in breast cancer is characterized by change in the tumor biology, which leads to discordance in receptor status. Repeat biomarker studies at metastatic recurrence is warranted, especially if treatment plans include hormone and targeted therapy.

## INTRODUCTION

Brest cancer represents the most common cancer in Indian women, with an age-adjusted rate of 25.8 cases per 100,000 women and a mortality rate of 12.7 per 100,000 women.^1^ Incidence of metastatic breast cancer (MBC) has been reported to be approximately 5% to 25% from various centers in India.^2^ In a previous study from our center, we found that 26.5% of patients were diagnosed with MBC on initial presentation and 22% developed metastasis at recurrence.^3^ In India, most patients with MBC receive treatment at tertiary care centers because of a lack of services at primary and secondary centers. MBC treatment depends on patients’ hormone and human epidermal growth factor receptor 2 (HER2/*neu*) status. Receptor discordance between primary and recurrent breast cancer in estrogen receptor (ER), progesterone receptor (PR), and HER2/*neu* has been documented to be approximately 12% to 35%, 30% to 50%, and 5% to 15%, respectively, in Western literature, but data from developing countries is limited.^4-9^ The 4th European School of Oncology–European Society of Medical Oncology (ESO–ESMO) international consensus guidelines for advanced breast cancer recommend repeat biopsy and documentation of any discordance in receptor status at metastasis, but this is yet to find a place in the national Indian Council of Medical Research guidelines.^10,11^

Change in receptor status as an aid to decide additional management and prognosis in MBC must be tested in a randomized controlled trial. From the Indian perspective—where the impact of the disease is high and patients present with advanced stage disease with increasing incidence—an epidemic of breast cancer in the near future may result.^12^ In such a situation, the best use of health care resources—with newer therapies and a multidisciplinary treatment approach—serves as an indispensable tool for the evidence-based management of MBC. Outside the context of public health care, Indian medical oncologists also need to address individual patient priorities and their rights for current standard management. With the background of the above facts and the lack of recommendations in Indian management guidelines, we designed an ambispective cohort study to compare hormone receptors—ER and PR—and HER2/*neu* receptor at baseline and first metastasis. To our knowledge, this is the first work to document receptor status change in patients from India.

## METHODS

We designed an ambispective study with the aim of documenting discordance in biomarker (ER, PR, and HER2/*neu*) status in patients with breast cancer (stage I, II, and III per American Joint Committee on Cancer, 7th edition) who develop metastasis on follow-up and the resultant change in therapy. When patients develop metastasis, there may be only distant metastasis or distant metastasis with locoregional recurrence. In the current study, we documented biomarker status at baseline from primary breast lesion and at first metastasis from the locoregional or metastatic site (Table 1). This study was conducted at All India Institute of Medical Science (New Delhi, India). We reviewed the records of our patients who developed metastasis between January 2013 and August 2018, recruiting them in a prospective (n = 65 patients) and retrospective manner (n = 38 patients). Ethical clearance was provided by the institute ethics committee (reference No. IECPG-539/26.10.2016), and informed consent was obtained from patients who were recruited prospectively. During the study period, 205 patients with breast cancer developed metastasis at recurrence. Of these patients, 103 were considered for analysis. Approximately 102 patients were excluded as a result of various reasons as given in Figure 1. Patients who had bone-only metastasis without any locoregional recurrence, impending organ failure (visceral crisis), and poor performance status were not subjected to rebiopsy. Patients who were diagnosed with recurrence using fine-needle aspiration cytology, fluid cytology, and incomplete clinical and pathologic details were not included in this study (Fig 1). However, patients with bone-only metastasis and locoregional recurrence were included in our study. A tissue for biomarker analysis was obtained using Tru-cut biopsy or surgical resection at recurrence from various sites as given in Table 1.

**TABLE 1:**
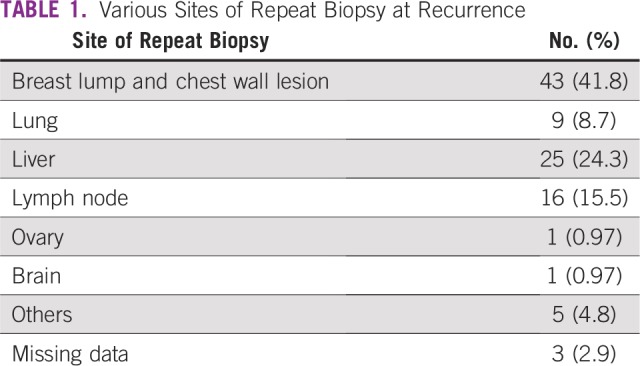
Various Sites of Repeat Biopsy at Recurrence

### Pathology: Immunohistochemistry and Fluorescence In Situ Hybridization

Hormone and HER/2neu were documented using the standard immunohistochemistry method and results were interpreted by two pathologists. Allred scoring was used for reporting ER/PR receptor status, and we recorded the percentage of cells showing ER positivity and intensity. A score of 3+ was considered positive.^13^ HER2/*neu* receptor was documented per the ASCO/College of American Pathologists guidelines.^14^ A score of 3+ was considered positive and 2+ was considered equivocal. All 2+ results of HER2/*neu* were confirmed using the fluorescence in situ hybridization method per standard ASCO/College of American Pathologists guidelines.^14^ Histologic type was assessed according to the WHO standard.

### Treatment Protocol

Our institute protocol of combination chemotherapy for primary tumor management is anthracycline–taxane or taxane–platinum-based chemotherapy—four-cycle fluorouracil 600 mg/m^2^ plus epirubicin 75 mg/m^2^ plus cyclophosphamide 600 mg/m^2^ followed by four-cycle docetaxel 85 mg/m^2^ (or docetaxel 75 mg/m^2^ + carboplatin area under the curve 6). Those patients who underwent modified radical mastectomy received adjuvant radiotherapy 50 Gy/25 fractions over 5 weeks when indicated. Those who underwent breast-conserving surgery received adjuvant radiotherapy 50 Gy/25 fractions with 16-in-8 fraction boost over 2 weeks. Endocrine therapy (ET), 10 years tamoxifen, or 5 years aromatase inhibitor was administered to hormone receptor (HR) –positive patients and 1 year of trastuzumab for HER2/*neu*-positive patients. Institute protocol for MBC management is a single agent or combination chemotherapy, ET, cyclin-dependent kinase 4/6 (CDK4/6) inhibitor, and targeted therapy based on receptor status.

### Statistical Analysis

Data documented from hospital records included age, sex, menopausal status, side, histology, biomarker status (ER, PR, and HER2/*neu*), stage, and site of metastasis. Nominal data presented as number and percentage and continuous data presented as medians and range. Data were analyzed using STATA software (version 13; STATA, College Station, TX; Computing Resource Center, Santa Monica, CA).

## RESULTS

### Patient Characteristics

Clinical and pathologic characteristics at baseline are listed in Table 2. Median age of presentation was 47 years (range, 26 to 72 years). The most common histology was infiltrating ductal carcinoma. The majority of patients presented with locally advanced disease (stage I, 6.8%; stage II, 32%; and stage III, 61.2%; on the basis of American Joint Committee on Cancer, 7th edition).

**TABLE 2:**
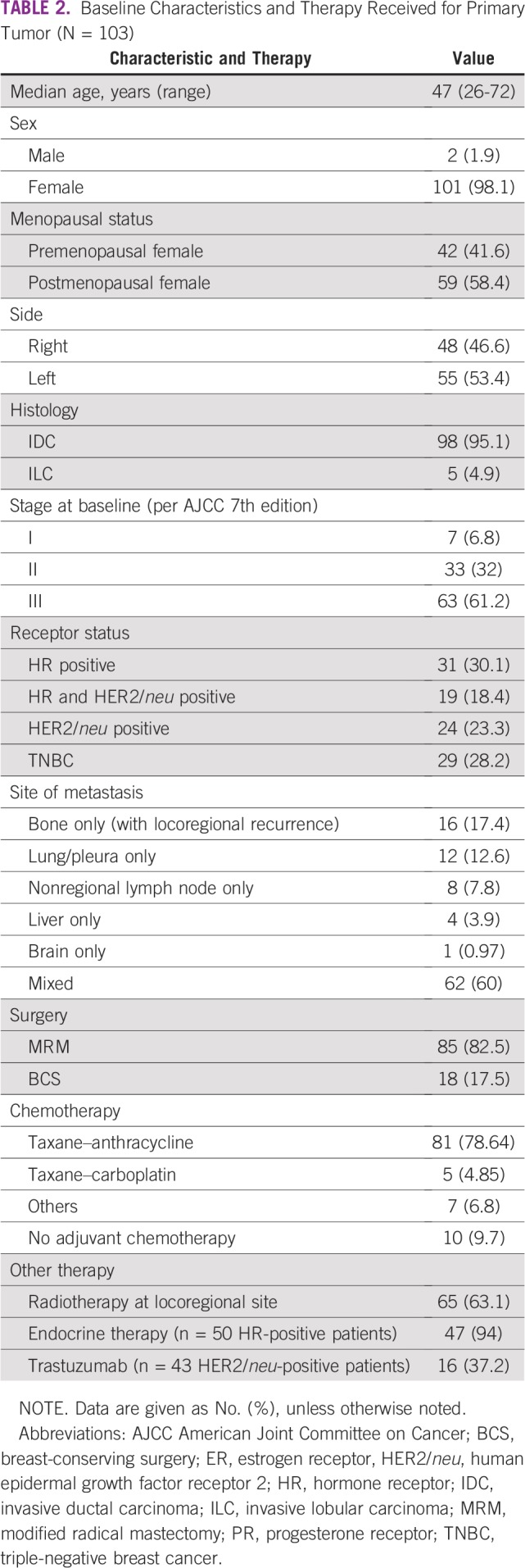
Baseline Characteristics and Therapy Received for Primary Tumor (N = 103)

In our cohort, 41 patients (40%) had single-site metastasis, including bone (16 patients; 15.5%), lung (12 patients; 11.7%), nonregional lymph node (eight patients; 7.8%), liver (four patients; 3.9%), and brain (one patient; 0.97%). More than one site of metastasis was present in 62 patients (60%; Table 2). The most common sites of metastasis were visceral (lung and liver) followed by bone, nonregional lymph node, skin, and brain. Baseline biomarker status demonstrated that 31 patients (30.1%) were HR positive, 19 (18.4%) were both HR and HER2/*neu* receptor positive, 24 (23.3%) were HER2/*neu* receptor only positive, and 29 (28.2%) were triple-negative breast cancer as listed in Table 2. For primary tumor management, approximately 85 patients (82.5%) underwent modified radical mastectomy, 18 patients (17.5%) underwent breast-conserving surgery, 81 patients (78.64%) received taxane–anthracycline-based chemotherapy, 47 patients (94% HR positive and 45.6% of the whole cohort) received ET, and 16 patients (37.2% HER2/*neu* positive and 15.5% of the whole cohort) received trastuzumab. Approximately 65 patients (63.1%) received radiotherapy (Table 2).

### Discordance in Biomarkers at the Time of Metastasis

In our cohort of 103 patients, discordance was present in 22 (21.3%) for ER, 30 (29.1%) for PR, and 16 (15.5%) for HER2/*neu* receptor status. Approximately eight patients (7.8%) had positive to negative ER status transition and 14 (13.6%) negative to positive. Whereas 22 patients (21.4%) had positive to negative PR status, eight patients (7.8%) had negative to positive PR status. Overall, nine patients (9.2%) had positive to negative HR (ER or PR or both) and 12 negative to positive. Approximately seven patients (6.8%) had positive to negative HER2/*neu* receptor status and nine (8.7%) negative to positive (Table 3).

**TABLE 3:**
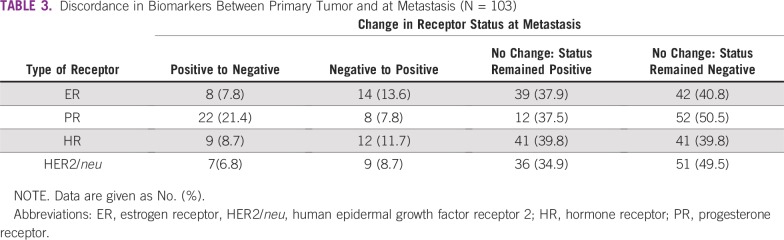
Discordance in Biomarkers Between Primary Tumor and at Metastasis (N = 103)

### Therapeutic Impact of Discordance in Receptor Status

Per the 4th ESO–ESMO international consensus guidelines for advanced breast cancer, all patients who were receptor positive at least once at baseline or at metastasis should be administered ET or targeted therapy according to receptor status.^10^ Considering the above guidelines, 62 patients were eligible for ET and 51 patients were eligible for targeted therapy. Among eligible patients, 41 patients (66.1%) received ET and 24 (47%) received targeted therapy (Table 4). Of 12 patients who were negative to positive for HR, nine received ET, and of nine patients who were positive to negative for HR, three received ET. Of nine patients who were negative to positive for HER2/*neu*, three received targeted therapy, and of seven patients who were positive to negative for HER2/*neu*, three received targeted agent. In conclusion, of 21 patients who had receptor gain (HR, n = 12; HER2/*neu*, n = 9), we could modify treatment in 12 patients (ET, n = 9; targeted therapy, n = 3), per new receptor status.

**TABLE 4:**
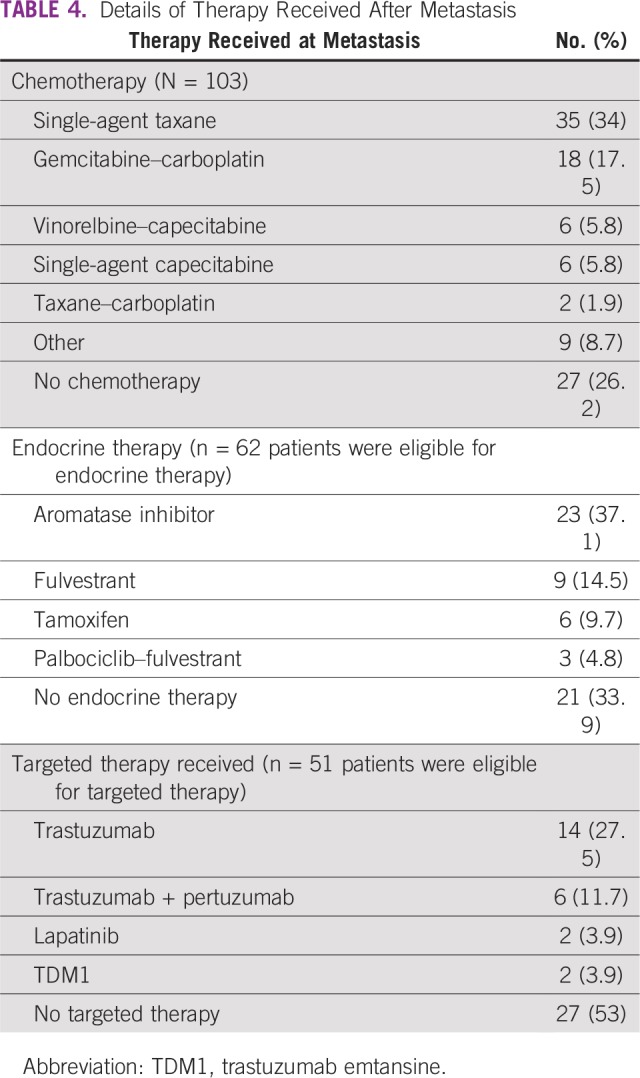
Details of Therapy Received After Metastasis

## DISCUSSION

MBC is a heterogeneous disease and most patients present with advanced stage disease in India. There are multiple mechanisms proposed for the discordance in biomarkers: a switch in the biology of the disease; sampling error in focally receptor-positive cancers; limited reproducibility of receptor assays; heterogeneous tumors with different clinical characteristics, disease course, and responses to specific treatment; and previous treatment that may change in receptor status.^15-19^ A systematic approach for the management of MBC is essential to improve patient outcomes and prognoses. Data from previous Western studies suggest that the discordance of ER, PR, and HER2/*neu* between primary and metastatic disease may be clinically relevant. There is no such study published from India. We designed an ambispective study with the intent of documenting change in biomarker status from baseline to first metastasis and to observe subsequent change in therapy. A summary of our study and various previous studies from Western countries is documented in Table 5. Precise comparison of our present study with previous studies is difficult because all parameters were not reported in other studies. In this study, an effort was made to evaluate trends in HR change at metastasis for patients who present with breast cancer in India.

**TABLE 5:**
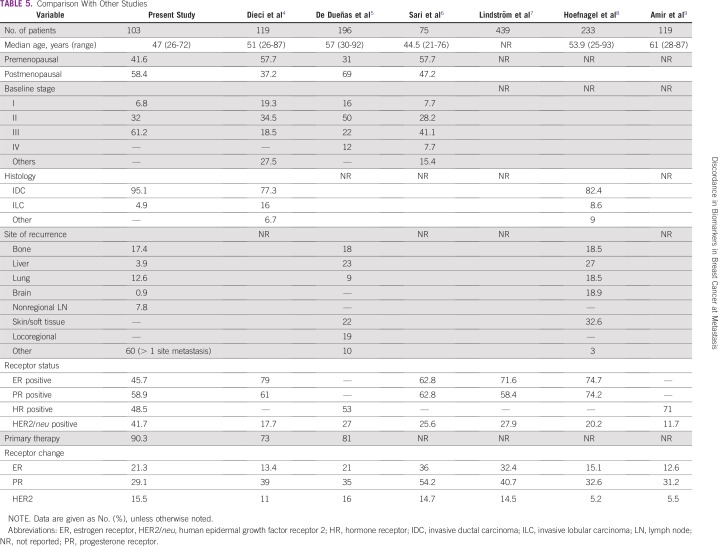
Comparison With Other Studies

We found that the discordance was most common in PR, followed by ER and HER2/*neu* receptor. Results showed a similar trend, but in relatively different proportions from that documented in previous studies. We observed that the median age of presentation was 47 years, which is 5 to 10 years less when compared with other studies, with the exception of the study by Sari et al. Most of our patients presented in advanced stage: 61.2% of patients presented in stage III, whereas only 32% were stage II, and 6.8% were stage I. Of patients, 40% had single organ involvement, whereas the remaining 60% had multiorgan involvement. This indicates that disease burden is substantially higher in patients at the time of metastasis. Compared with other studies, the proportion of HR-positive breast cancer (48.5%) is lower, whereas the proportion of HER2/*neu*-positive breast cancer is higher in this study (41.7%). This helps to explain why the discordance in HR status (at metastasis) is relatively less and discordance in HER2/*neu* receptor status is relatively greater in our cohort. In a study by De Dueñas et al,^5^ different results were reported in central and local laboratories. Receptor changes recorded were higher at the local level compared with the central laboratory (change in HER2/*neu*, 16% *v* 3%; change in ER, 21% *v* 13%; and change in PR, 35% *v* 28%, respectively). This highlights the importance of a standardized technique for assay and reporting of receptor status. In another study by Lindström et al,^7^ receptor change was reported at multiple relapses. This indicates that breast cancer is a heterogeneous disease, and metastatic disease evolution is associated with a change in receptor level after each progression. Receptor loss is associated with decreased survival as documented by Dieci et al, whereas Amir et al documented no difference in survival.^4,9^ The impact of receptor discordance on survival still is not conclusive and prospective studies are required to answer this question. Hoefnagel et al^8^ reported that the most common discordance is associated with PR, as documented in other studies. We found that all published studies had noted the importance of receptor status documentation at metastasis, despite varying results.

Documentation of change in receptor status at disease progression may help to improve disease management and patient care. In the current study, many of our patients did not receive targeted therapy and CDK4/6 inhibitor as a result of financial constraints. In patients with HER2/*neu*-positive disease, only 37.2% received trastuzumab for primary therapy, whereas 47% patients received targeted therapy for metastasis. Approximately 14.5% and 4.8% of HR-positive patients received fulvestrant and CDK4/6 inhibitor, respectively. This indicates that many of our patients were not able to afford costly newer therapies for the treatment MBC; however, there are many upcoming patient assistance programs from the government of India and many nongovernment organizations. For instance, the prime minister relief fund for patients with cancer, Ayushman Bharat scheme, free-of-charge mediation for patients below the poverty line, and free-of-charge necessary chemotherapy medicine and hormonal agents (tamoxifen and anastrozole) for patients visiting government hospitals of India, such as All India Institute of Medical Science.^20,21^ With the help of these assistance programs and a decrease in the cost of trastuzumab compared with the price 5 years ago, we expect that these proportions will improve in the near future. The current international guideline for the treatment of advanced breast cancer recommends rebiopsy in advanced breast cancer at first metastasis, but existing Indian Council of Medical Research guidelines 2016 do not mention the importance of repeat biopsy.^10,11^ The present study may serve to inform and fill the lack of an existing national guideline. We do not suggest reallocating health care resources for costly newer therapy in patients with MBC, but to rationalize the use of the best available therapeutic options.

The strength of our study is its ambispective design coupled with the receptor status documentation on the initial visit and with metastasis. We have linked HER2/*neu* 2+ status with fluorescence in situ hybridization analysis. A relative weakness of our study is the collection of data for few of the cohort patients in a retrospective manner. This study is not powered for survival analysis, and a large prospective multicenter study is required to answer this question. The current study may serve to challenge many centers in India, where routine rebiopsy is not performed at patient presentation with metastatic recurrence.

To our knowledge, this is the first study from a developing country to report receptor status change in MBC; however, this study does not address whether to withdraw ET or targeted therapy after the loss of receptor status. As a result of tumor heterogeneity, the biopsy sample may not show receptor positivity at one site, but other sites may be positive. Therapy withdrawal may deprive the patient of the benefit of targeted agents. Fluoroestradiol positron emission tomography/computed tomography may address this question of assessing receptor status in all metastatic sites in the near future. Therapy withdrawal may deprive the patient of the benefit of ET and targeted therapy. From the 4th ESO–ESMO international consensus guidelines for advanced breast cancer, “If the results of tumor biology in the metastatic lesion differ from the primary tumor, it is currently unknown which result should be used for treatment decision making. Since a clinical trial addressing this issue is difficult to undertake, we recommend considering the use of targeted therapy (endocrine therapy and/or anti HER2 therapy) when receptors are positive in at least one biopsy, regardless of timing.”^10^^(p4)^ We followed the same principle and offered appropriate therapy for eligible patients; however, as stated, only approximately one half of patients received targeted agents, and 4.8% patients received CDK4/8 inhibitor.

In light of the above findings, we recommend that patients with newly diagnosed metastatic disease undergo repeat biopsy, which will not only help to confirm the diagnosis of recurrence but also will also allow for a re-examination of receptor status. However, we acknowledge that there is an unmet need for prospective studies from developing countries verifying receptor status in new metastases and the resultant changes in survival.

## References

[1] Malvia S Bagadi SA Dubey US et al Epidemiology of breast cancer in Indian women Asia Pac J Clin Oncol 13 289 295 2017 28181405 10.1111/ajco.12661

[2] Gogia A Raina V Deo SVS et al Triple-negative breast cancer: An institutional analysis Indian J Cancer 51 163 166 2014 25104201 10.4103/0019-509X.138275

[3] Sharma M Gogia A Deo SVS et al Profile of metastatic breast carcinoma: Single center experience from a developing country J Clin Oncol 36 15s 2018 suppl; abstr e13102

[4] Dieci MV Barbieri E Piacentini F et al Discordance in receptor status between primary and recurrent breast cancer has a prognostic impact: A single-institution analysis Ann Oncol 24 101 108 2013 23002281 10.1093/annonc/mds248

[5] de Dueñas EM Hernández AL Zotano AG et al Prospective evaluation of the conversion rate in the receptor status between primary breast cancer and metastasis: Results from the GEICAM 2009-03 ConvertHER study Breast Cancer Res Treat 143 507 515 2014 24414130 10.1007/s10549-013-2825-2 PMC3907670

[6] Sari E Guler G Hayran M et al Comparative study of the immunohistochemical detection of hormone receptor status and HER-2 expression in primary and paired recurrent/metastatic lesions of patients with breast cancer Med Oncol 28 57 63 2011 20099049 10.1007/s12032-010-9418-2

[7] Lindström LS Karlsson E Wilking UM et al Clinically used breast cancer markers such as estrogen receptor, progesterone receptor, and human epidermal growth factor receptor 2 are unstable throughout tumor progression J Clin Oncol 30 2601 2608 2012 22711854 10.1200/JCO.2011.37.2482

[8] Hoefnagel LD van de Vijver MJ van Slooten H-J et al Receptor conversion in distant breast cancer metastases Br Canc Res 12 R75 2010 http://breast-cancer-research.biomedcentral.com/articles/10.1186/bcr2645 10.1186/bcr2645 PMC3096964 20863372

[9] Amir E Clemons M Purdie CA et al Tissue confirmation of disease recurrence in breast cancer patients: Pooled analysis of multi-centre, multi-disciplinary prospective studies Cancer Treat Rev 38 708 714 2012 22178456 10.1016/j.ctrv.2011.11.006

[10] Cardoso F Senkus E Costa A et al 4th ESO-ESMO International Consensus Guidelines for Advanced Breast Cancer (ABC 4)† Ann Oncol 29 1634 1657 2018 30032243 10.1093/annonc/mdy192 PMC7360146

[11] India Against Cancer ICMR consensus documents for cancer management http://cancerindia.org.in/icmr-consensus-documents-for-cancer-management/

[12] Shetty P India faces growing breast cancer epidemic Lancet 379 992 993 2012 22432152 10.1016/s0140-6736(12)60415-2

[13] Harvey JM Clark GM Osborne CK et al Estrogen receptor status by immunohistochemistry is superior to the ligand-binding assay for predicting response to adjuvant endocrine therapy in breast cancer J Clin Oncol 17 1474 1481 1999 10334533 10.1200/JCO.1999.17.5.1474

[14] Wolff AC Hammond MEH Allison KH et al Human epidermal growth factor receptor 2 testing in breast cancer: American Society of Clinical Oncology/College of American Pathologists Clinical Practice Guideline focused update J Clin Oncol 36 2105 2122 2018 29846122 10.1200/JCO.2018.77.8738

[15] Pusztai L Viale G Kelly CM et al Estrogen and HER-2 receptor discordance between primary breast cancer and metastasis Oncologist 15 1164 1168 2010 21041379 10.1634/theoncologist.2010-0059 PMC3227913

[16] Bertos NR Park M Breast cancer: One term, many entities? J Clin Invest 121 3789 3796 2011 21965335 10.1172/JCI57100 PMC3195465

[17] Wu JM Fackler MJ Halushka MK et al Heterogeneity of breast cancer metastases: Comparison of therapeutic target expression and promoter methylation between primary tumors and their multifocal metastases Clin Cancer Res 14 1938 1946 2008 18381931 10.1158/1078-0432.CCR-07-4082 PMC2965068

[18] Pectasides D Gaglia A Arapantoni-Dadioti P et al HER-2/neu status of primary breast cancer and corresponding metastatic sites in patients with advanced breast cancer treated with trastuzumab-based therapy Anticancer Res 26 647 653 2006 16739334

[19] Mittendorf EA Wu Y Scaltriti M et al Loss of HER2 amplification following trastuzumab-based neoadjuvant systemic therapy and survival outcomes Clin Cancer Res 15 7381 7388 2009 19920100 10.1158/1078-0432.CCR-09-1735 PMC2788123

[20] India Against Cancer Financial aid and resources http://cancerindia.org.in/financial-aid-and-resources/

[21] National Portal of India Ayushman Bharat—National Health Protection Mission https://www.india.gov.in/spotlight/ayushman-bharat-national-health-protection-mission

